# From Natural Towards Representative Decision Making in Sports: A Framework for Decision Making in Virtual and Augmented Environments

**DOI:** 10.1007/s40279-023-01884-3

**Published:** 2023-09-01

**Authors:** Tim Janssen, Daniel Müller, David L. Mann

**Affiliations:** 1 Department of Performance Analysis & Technology, Royal Dutch Football Association (KNVB, Koninklijke Nederlandse Voetbalbond), Zeist, The Netherlands; 2 Department of Human Movement Sciences, Faculty of Behavioural and Movement Sciences, Amsterdam Movement Sciences and Institute Brain and Behaviour Amsterdam (iBBA), Vrije Universiteit Amsterdam, Amsterdam, The Netherlands

## Abstract

Decision making is vital in complex sporting tasks but is difficult to test and train. New technologies such as virtual and augmented reality offer novel opportunities for improving decision making, yet it remains unclear whether training gains using these new approaches will improve decision making on-field. To clarify the potential benefits, a clear conceptualization of decision making is required, particularly for invasive team sports such as football, basketball and field hockey, where decisions are complex with many possible options offered. Therefore, the aim of this position paper is to establish a framework for the design of virtual and augmented environments that help invasive team sport athletes to train their decision-making capacities. To achieve this, we propose a framework for conceptualising ‘natural’ decision making within the performance environment in invasive team sports that views decision making as a continuous cyclical process where the ball carrier interacts with teammates to create ‘windows of opportunity’, and where skilled decision makers often delay decisions to create time, and in turn new opportunities, rather than necessarily selecting the first option available to them. Within the framework, we make a distinction between decision making and anticipation, proposing that decision making requires a series of on-going anticipatory judgments. Based on the framework, we subsequently highlight the consequences for testing and training decision making using virtual and augmented reality environments, in particular outlining the technological challenges that need to be overcome for natural decision making to be represented within virtual and augmented environments.

## Key Points

**Table:** 

Decision making in complex team sports is conceptualized as a cyclical process involving interactions between the ball carrier and their teammates to create ‘windows of opportunity’.
Current approaches for the testing and training of decision making in invasive team sports (e.g. video footage and virtual/augmented reality) typically lack the interactions between teammates that form a vital element of decision making.
We set out four design principles (representative interactions, movements, viewpoint and scenarios) developed to create decision-making scenarios in virtual reality that are capable of improving on-field decision making.

## Introduction

Decision making in invasive team sports is complex given the number of players on the field and the unpredictable way that patterns of play evolve. Invasive team sports are those such as basketball, rugby and netball where two teams moving in opposite directions seek to advance forward into their opponent’s territory to score [1]. At almost any moment, players in invasive sports can halt or change their course of action, making every situation complex, unique, and unpredictable. Take field hockey, where the player in ball possession has—among other options—the possibility to shoot, pass, dribble or evade their opponent, all while their teammates move to create passing options or space, and opponents try to shut down those options and constrain space [2]. This results in the ball carrier and their teammates co-conspiring to out-maneuver their opponents to create moments where teammates are spatially ‘open’ as passing options for discrete periods of time. As a result, the ball carrier has a role in both creating those opportunities and in exploiting them.

Decision making can be conceptualized using different theoretical perspectives, often leading to very different conclusions about the conditions necessary to test and train decision making. The representational approach is exemplified by the fundamental work of de Groot [3], who found that expert chess players could better recall the locations of chess pieces when positioned meaningfully on a board in a game situation. The better recall led to the conclusion that experts are better able to use mental representations to recall information from similar situations, and that this information can then be used to generate better decisions about the most appropriate action to take when placed in a similar scenario. Building on this, invasive team-sport athletes have been shown to be better able to recall the locations of players on a field of play [4–6] and are better able to make decisions about the most appropriate action to take when placed in match-specific situations [7, 8]. Using this approach, decision making in invasive team sports has often been tested using an occlusion paradigm where players watch video footage of an emerging pattern of play and decide what action they would take when the footage is occluded at an a priori defined point in time (i.e. the screen goes black or freezes) [7, 8]. Results using the occlusion paradigm typically show that experts in invasive team sports make superior and/or faster decisions [5, 8]. These experimental paradigms possess high scientific control; however, questions remain about the degree to which they test the type of skills necessary to make natural decisions on-field. In particular, participants typically do not move while making the decisions using these paradigms, negating both the embodied nature of decision making [9] and the decision-maker’s role in creating opportunities. Moreover, the requirement to make a decision at a discrete moment in time—and often to respond as quickly as possible—negates the ability of the decision maker to exploit time to wait for a more appropriate decision to emerge. As a result, concerns remain about the suitability of the occlusion paradigm not only for testing, but also for training decision making given that some presumably important aspects of decision making are not required for success.

The ecological dynamics approach advocates that decisions emerge as a result of the interaction between the performer and environment, and therefore that optimal decisions will differ according to the action capabilities of the performer and the opportunities the performer perceives for action [10–12]. Accordingly, every decision-making scenario is considered to be unique and therefore the correct or ‘ideal’ outcome does not emerge through a mental recollection of similar scenarios encountered previously.

It remains challenging to test decision making in a consistent and repeatable manner while remaining faithful to the ecological dynamics approach. In particular, testing should allow the performer to interact with their environment. Moreover, a ‘correct’ decision should be viewed relative to the action capabilities of the performer. Crucially, tests should be ‘representative’ of the natural task they seek to represent, and should incorporate the perceptual variable(s) offered in the natural environment [13]. In all likelihood, this results in test conditions that differ across participants, because no two scenarios are likely to ever be the same given that the participant will move in their own unique way to generate perceptual information and interactions as they do on-field.

The debate about how decision making should be conceptualized leads to confusion about whether new training designs will lead to on-field gains in decision making. For example, virtual-reality environments (encompassing virtual, augmented and mixed reality) provide a form of complementary training that is appealing to many sport organizations, both for supplementing sport training more broadly and for offering training opportunities while recovering from injury [14, 15]. A number of position papers have already addressed the potential for virtual reality to improve motor and/or sport performance more broadly [15–20], with empirical evidence now emerging to confirm that improvements in motor performance are indeed possible using those technologies (e.g. [21]). More specifically for decision making, there is also evidence to show that virtual environments can be used to improve decision making in other fields of expertise where decisions are typically de-coupled from actions (e.g. medical decision making [22]), and in in some discrete tasks in interceptive hitting sports (e.g. baseball batting [21]). However, the degree to which natural decision making can be improved using virtual reality remains particularly unknown in invasive team sports where the decision maker seeks to interact with their team mates and opponents. In particular, it is our contention that the current technologies available using virtual reality are not yet sufficiently developed for those environments to present these interactions and therefore for them to realize their potential to improve on-field decision making. Nonetheless, rapid technological developments offer promise for these shortcomings to be overcome in the very near future (e.g. see [15, 23]). As a result, clear guidelines with respect to the necessary requirements for these environments are required.

The aim of this paper is to establish a framework for the design of virtual environments capable of training the on-field decision-making performance of invasive team-sport athletes. To achieve this aim, we outline our conceptualization of decision making in invasive team sports within their natural performance environment (what we refer to in this paper as ‘natural decision making’). We then apply the framework to establish the design principles necessary for developing representative virtual environments that would be suitable for testing and training natural decision making.

## A Framework for Natural Decision Making in Invasive Team Sports

**Fig. 1:**
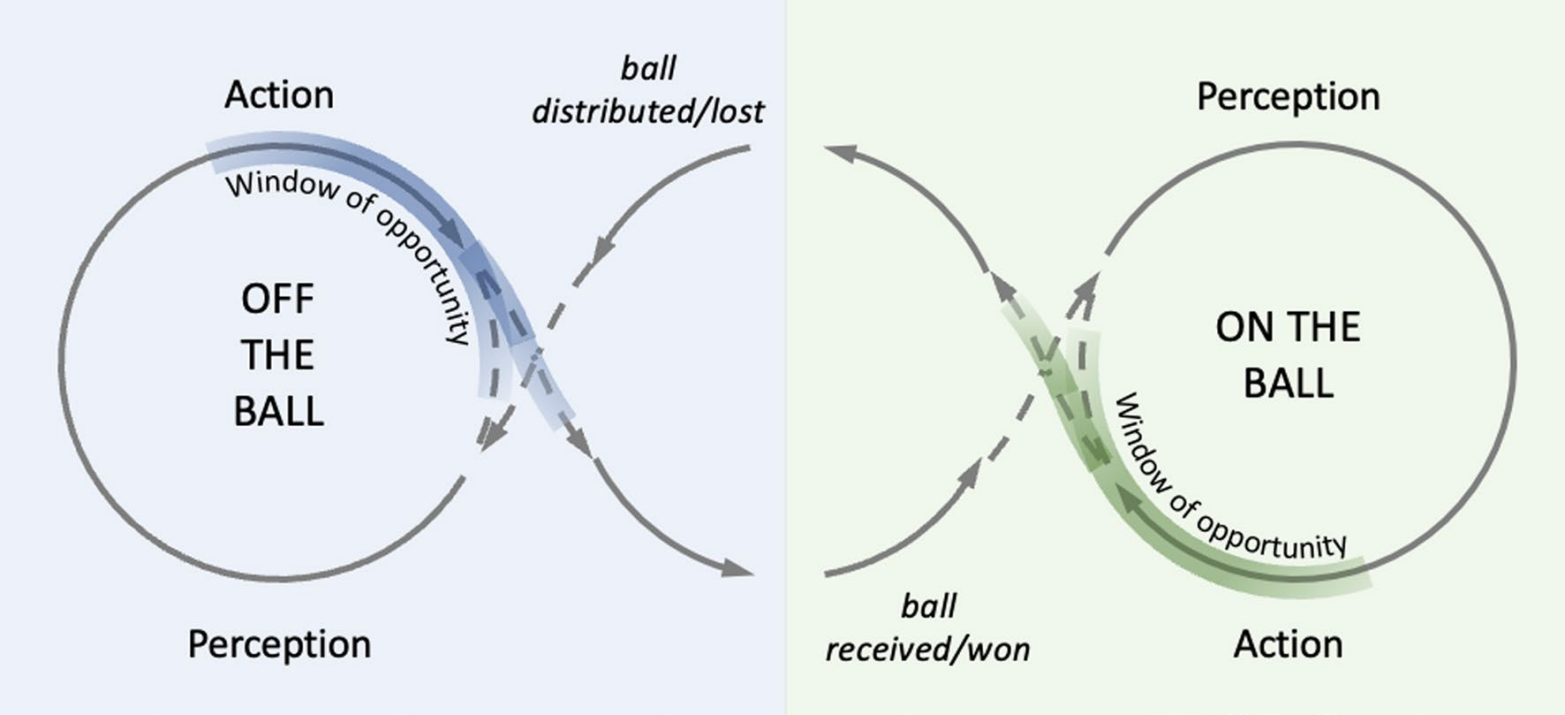
A framework for natural decision-making in an invasive team sport. The right side of the figure shows the actions of the attacking player who has ball possession (i.e. is ‘on the ball’), they are in a continuous perception–action cycle where they co-conspire with other teammates (and avoid opponents) to create opportunities for them to pass, evade their opponent or shoot (‘windows of opportunity’). If they do not take the opportunity, they maintain possession and the cycle continues. The left side of the figure shows the actions of the teammates and opponents who are ‘off the ball’. Those players are also in a perception–action cycle where they act to create (passing) opportunities for the teammate in possession and the defenders act to prevent those opportunities

A player with ball possession in an invasive team sport is not necessarily required to make a single decision in isolation, but rather is often faced with a continuous cycle of decisions influenced by the interaction of their own movements with those of their teammates and opponents. Moreover, those teammates and opponents without the ball also face a continuous cycle of decisions: attacking teammates seek to create opportunities for the player with the ball; and defending opponents try to close those opportunities. Figure 1 shows a framework that seeks to reflect the cyclical nature of the decisions made by the ball carrier (right side of figure), and their teammates and opponents (left side of figure).

The right side of Fig. 1 represents when a player of interest is in ball possession. When they receive the ball, they move into a perception–action cycle where they perceive-to-act and act-to-perceive. The ball carrier co-conspires or interacts with their teammates to create ‘windows of opportunity’ where it is feasible for the ball carrier to successfully pass the ball to their teammate(s) without it being intercepted by their opponents (or even possibly to run with the ball or to shoot at goal). The ball carrier may take the first opportunity that arises, as would be suggested by the ‘take-the-first’ heuristic, a strategy which proposes that better and more consistent decisions are made when simply taking the first option that arises because it reduces the options generated [24, 25]. Alternatively, the decision maker might neglect that first option and instead wait for a potentially better option to emerge. In that case, they remain in the perception–action cycle to explore and/or create additional windows of opportunity. Crucially, the framework conceptualizes two important ideas that are sometimes neglected in typical views of decision making. First, the perception–action cycle encapsulates the important role that the ball carrier has in creating opportunities for action. The ball carrier is not necessarily a passive observer waiting for opportunities to arise, but rather they move to create opportunities by interacting with their teammates. Most existing tests of decision making in invasive team sports miss this important element of expertise because the movements of the teammates seen in the scenario do not respond to the actions of the observer (i.e. for tests when viewing video footage, e.g. [8, 26–28]; 360-degree video, e.g., [29]; and some forms of non-interactive virtual reality, e.g. [30]). Second, the cyclical nature of the framework reflects our observation that excellent decision makers do not necessarily take the first option available to them [24, 25], but rather will wait and rely on their own technical and perceptual skills to create new, potentially better options. This highlights the point that ‘windows of opportunity’ come and go, and that athletes are not necessarily bound to make decisions at discrete moments in time. Rather, they interact with others and can wait until an option emerges that they are happy to accept. 

The left side of Fig. 1 reflects the crucial decision-making role that players *without the ball* have in creating or closing windows of opportunity. Attacking teammates without the ball must make their own decisions about which direction they should move to create potential opportunities for the ball carrier (e.g. for passing or shooting). Teammates must take into consideration their own action capabilities, relative to those of their defender(s), in addition to the capabilities of their other teammates (particularly the ball carrier). The teammate too perceives-to-act and acts-to-perceive to create windows of opportunity for the ball carrier, or even for the teammate most likely to be the next ball carrier. If the opportunity is not taken, the cycle continues.

The left side of Fig. 1 also accounts for the decision-making role of defensive players when seeking to close windows of opportunity and to regain ball possession. While defending, players close to the opponent in ball possession may try to prevent opportunities for the player to pass to a teammate [31], or to score. Defenders close to opponents without the ball maintain their position to cover spaces and retain defensive structures [31], or in some sports follow their opponents to prevent them from receiving the ball (e.g. hockey or football). While defending, players make series of decisions to minimize windows of opportunities for their opponents. This cycle continues until possession is regained by the defending team. The left side of the framework shown in Fig. 1 has been included to reflect the important role of off-the-ball decision making, an element of expertise that has received little attention so far in the scientific literature. We contend that off-the-ball decision making is a skill that requires greater attention from a research perspective, particularly given that coaches, when developing tactical game strategies, often focus on the decision making of off-the-ball players both in attack and defense. Focus on this area will help to better identify and recognize players who contribute to team performance through their off-the-ball behaviour.

### An Applied Example of Natural Decision Making

Here, we illustrate this framework using an example from football. The line-breaking pass is one of the most pertinent attacking actions in football because it allows the attacking team to advance the ball forward through the defensive ‘lines’ of their opponent (e.g. the midfield and defensive lines). A key goal of the ball carrier is often to attempt a line-breaking pass to a teammate positioned in between the defensive lines of their opponents [32].

**Fig. 2:**
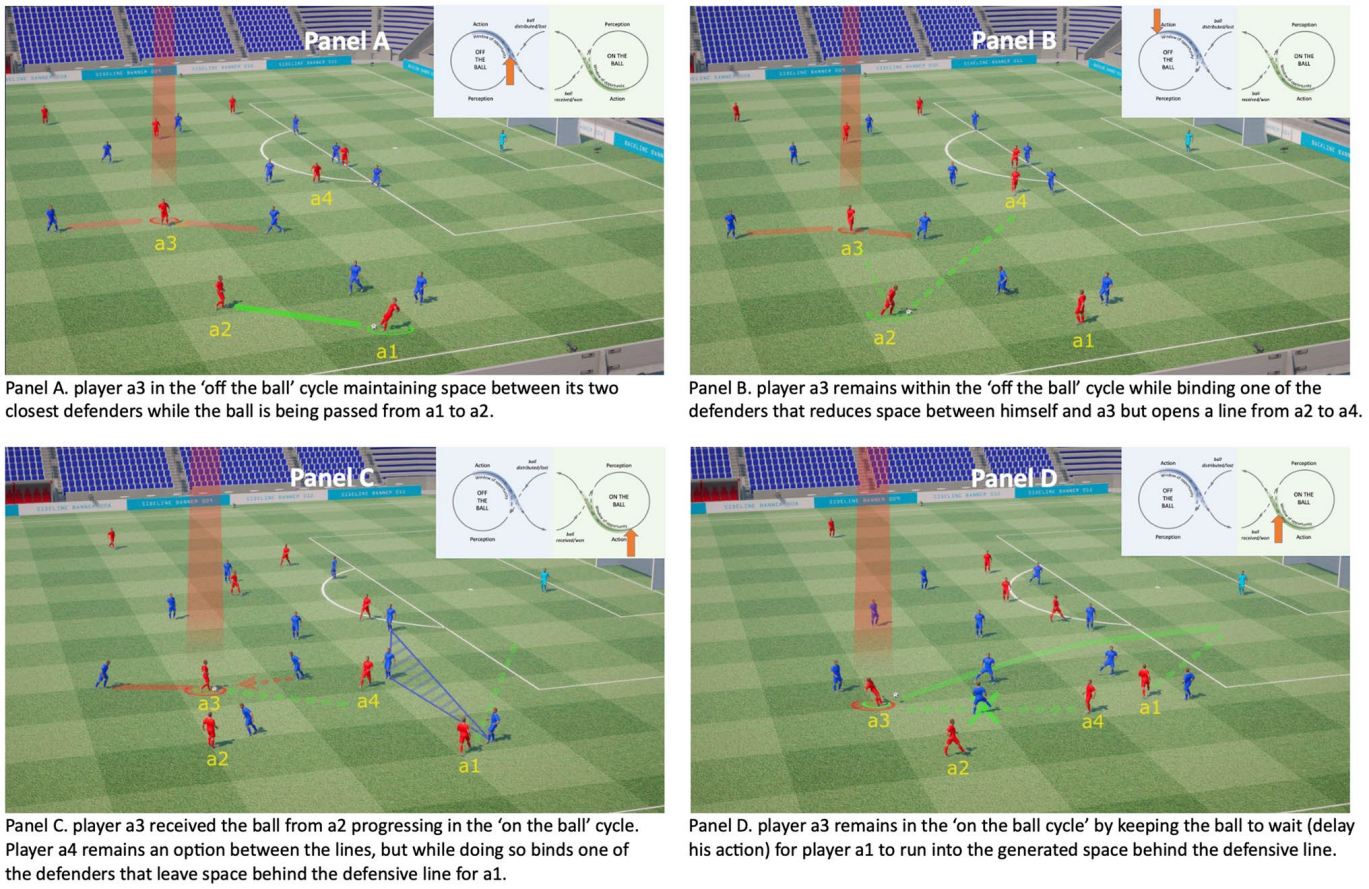
A typical sequence of play in football (~5 s duration) demonstrating a successfully delayed decision to pass behind the defensive line of the opponents. Labels a1–a4 indicate four players on the attacking team (red) while the defensive team (blue) employs a 4-4-2 defensive structure (attacking, midline, and defensive lines from left to right respectively in Panel **A**). In Panel **A**, the ball is passed from player a1 to a2, while player a3 is in the ‘off the ball’ cycle maintaining space between their two closest defenders and creating a passing opportunity for their teammates. In Panel **B**, player a3 remains in the ‘off the ball’ cycle, but has drawn their closest defender towards them, and in doing so opens a potential passing line from player a2 to a4. In Panel **C**, player a3 has now received the ball, progressing to the ‘on the ball’ cycle. Player a4 is now an obvious passing option for a3 to pass the ball to to advance the ball between the defensive lines. Instead of passing to a3, in Panel **D** a3 has remained in the ‘on the ball’ cycle by keeping the ball to wait for player a1 to run into space behind the defensive line into a more advantageous position. A delay in the decision led to a more advantageous outcome for the attacking team

Figure 2 shows an example of a line-breaking pass taken from an actual match (FC Barcelona vs Valencia) to illustrate key principles in our framework of decision making. FC Barcelona midfielders such as Andrés Iniesta, Sergio Busquets and Frenkie de Jong are particularly adept at and known for their skill in decision making and line-breaking passes. Panel A highlights four players on the attacking team (FC Barcelona; players a1–a4) who are interacting to create a goal-scoring opportunity: Players a1–a3 are positioned between the attacking and midlines of their opponents whereas player a4 is between their mid and defensive lines. Player a1 has ball possession, while player a3 has positioned himself to create a passing opportunity for a1, or even for a2 if he becomes the ball carrier.

In panel B, the ball has been passed to a2, and highlighted player a3 performs an off-ball movement in the backward direction. This movement maintains space between him and his defender, but in dragging his defender towards him, has created space for Player a4 to move ‘between the lines’. Player a4's defender now has to make a decision to either follow his direct opponent, while giving away space behind him, or to stay in the defensive line and leave a4 open as a free player with space between the lines. Already, this example highlights (1) the potentially crucial decision-making role that off-the-ball players have in creating opportunities, and (2) the vital nature of the interactions between players.

In panel C, a2 has taken the window of opportunity to pass to a3. By virtue of his own off-the-ball movements, a3 now has a window of opportunity to ‘take-the-first’ and pass to a4. This would constitute a successful between-the-lines pass. Nonetheless, a3 does not take this opportunity because he has anticipated the movements of a1 into the space generated behind the defensive line. In doing so, a3 needs to evaluate the action capabilities of a1, relative to his defender, to anticipate whether a pass to his teammate is likely to be successful. Panel D shows that a3 does take this latter option to pass to a1 into space behind the defensive line. In this scenario, players who act too quickly might pass the ball between the lines to a4. However, superior decision makers often delay their actions to wait for better options to emerge. Moreover, they have the technical skills necessary to execute these potentially more challenging actions. By including the decision-making cycles, our framework seeks to capture this capability to not simply take the first option that emerges, but rather to wait, and to either create new opportunities or to wait for better options to arise.

The example of the Barcelona mid-fielder's exquisite decision making in Fig. 2 highlights several important principles for designing a paradigm to test decision making. First, players can create opportunities through their own off-the-ball movements. This important element of decision making is missed by paradigms that do not require (or allow) participants to move. Second, as the example shows, players can attract their defenders towards them and create a passing line to one of their teammates. Any paradigm that lacks interactions (e.g. pre-recorded scenarios) would miss this important aspect of expertise given that the movements of the athlete being tested do not influence the movements of the players seen in the video. Genuine interactions are needed between the movements of the participant and their teammates and opponents to test the full extent of expertise in decision making. Third, players can place their team in a more favourable position by not taking the first option available, but instead can wait for a better option to emerge. Paradigms that require a decision to be made at a fixed moment in time (e.g. the occlusion paradigm) fail to pick up on this potentially crucial aspect of expertise.

### A Distinction Between Anticipation and Decision Making

Decision making in a sport context often requires an athlete to take a course of action based at least in part on what they anticipate their opponents and teammates will do in the near future. For instance, the decisions taken in the example shown in Fig. 2 were made possible by the players anticipating the likely movements of their teammates and opponents to see whether the team mates would make it into space before any direct opponents. Accordingly, successful judgements such as these about whether to pass require athletes to make a series of on-going anticipatory judgements about the future positions of their teammates and opponents. Here, we wish to highlight the important role that anticipation plays in decision making, and to emphasize the implications this distinction has for testing and training decision-making.

Anticipation in a sports context typically refers to the ability of an athlete to foresee what an individual opponent or teammate will do in the near future. It is eminently clear that skilled athletes are better able to anticipate action outcomes based on the kinematics of their opponents [33–35], though this effect has typically been demonstrated in one-on-one scenarios (e.g. receiving a serve in tennis or trying to save a penalty kick in football goalkeeping) and only occasionally in complex team scenarios (e.g. see [36]). A good example of a one-on-one scenario is the early work of Abernethy and Russell [33], who compared the anticipatory skill of expert and novice badminton players. Participants viewed video footage of an opponent that was occluded at a critical moment in the opponent’s stroke, with participants required to predict the landing position of the shuttle [33]. The results showed that experts were better able to anticipate the likely landing position based on the postural body cues of their opponent [33], and that the source of their advantage appeared to be their ability to pick up information from the opponent’s arm and racquet. These skilled players are then in turn presumably better able to decide where to position themselves on the court to intercept their opponent’s stroke [37, 38]. The consequence for athletes in invasive team sports is that they too need to make these anticipatory judgements to foresee where teammates and opponents are directed to execute successful actions such as passes and shots at goal.

Skilled athletes not only anticipate an opponent’s action outcomes based on postural cues, they also rely on contextual sources of information to anticipate outcomes [39, 40]. For instance, a tennis player may know that opponents—irrespective of who they are—are more likely to play cross-court shots from particular locations on the court than they are down-the-line shots [39]. Moreover, individual opponents are likely to have their own action preferences [41]. For instance, a particular opponent might have a preference to perform a specific type of tennis serve when under pressure or for a particular game score [42]. When translated to invasive team sports, actors may be able to anticipate that teammates will move in a given direction based on team strategies, or that a specific opponent will have an action preference to step off one foot rather than the other [43, 44]. These additional sources of contextual information are likely to assist athletes to anticipate the actions of opponents to better prepare themselves to make the most appropriate decision within a given scenario.

We contend that decision making in invasive team sports requires a series of on-going anticipatory judgements. In this sense, anticipation can be viewed as a key element or subset of decision making. In its simplest form this could necessitate, for instance, a footballer anticipating the actions of up to ten individual teammates and 11 opponents. However, in all likelihood, the future actions of each individual will depend at least in part on the movements of others (e.g. an attacker and their respective defender) and so skilled players are likely to anticipate the actions of dyads or even constellations/patterns of players rather than each as an independent individual [4].

If we accept the role that anticipation plays in decision making, then this holds significant implications for how decision making should be tested and trained. In particular, it has been shown that anticipatory judgements are more accurate when they are produced while performing an action (i.e. when there is perception–action coupling) than they are when producing a verbal or a simplified movement response [34]. Moreover, Dicks et al. [45] showed that behavioural patterns, such as the gaze patterns of football goalkeepers in the lead up to a penalty kick, differed when the goalkeepers were required to actually intercept the ball rather than just verbally respond. Collectively, these results support the idea that the best results for decision making are likely to be found when tested in situations where participants also make movements rather than verbal or simplified responses.

In support of the evidence from studies of anticipation, evidence also exists to show that performance in decision-making tasks can be improved when moving rather than making verbal judgements. Oudejans et al. [9] tested the ability of individuals to make decisions about whether there was a safe opportunity to cross a road between cars. They found that better crossing decisions were made while participants were walking towards the road than when standing still. This further supports the idea that, when in motion, humans may be better able to make contextually relevant decisions (see [20]). Clearly, there is good reason for the actions of a decision maker to be tested (and trained) wherever possible while producing the same type of movements they would be expected to make in the natural environment.

## Representative Decision Making in Virtual and Augmented Environments

New developments in virtual and augmented reality offer exciting prospects for improving the decision-making skill of invasive team sport athletes, but it remains unclear to what degree improvements in natural decision making should be expected using these methods. Virtual reality typically refers to immersive environments that are entirely computer generated, most often today presented using head-mounted displays. In contrast, augmented reality refers to when the natural environment is supplemented with computer-generated stimuli. Note that we refer to virtual and augmented reality collectively as ‘virtual’ reality, but the principles remain the same irrespective of whatever form of virtual, augmented, mixed or even ‘actual’ reality is being considered for testing and training. Virtual reality in all its forms is appealing for sport training because it offers the possibility to create custom scenarios that may be very challenging to reproduce on field, can be tailored to an athlete’s specific needs [20], can be standardized across athletes (e.g. for standardized tests), and in which athletes can train in their own time, i.e. without relying on the presence of other teammates and opponents [15].

As noted earlier, a number of position papers and reviews have already made a case for the potential to improve general on-field sport performance as a result of training in virtual reality (e.g. [15–20]). The early empirical evidence appears supportive, with a series of studies showing that athletes enjoy using virtual reality [46], that motor tasks when performed in virtual reality are typically representative of those performed naturally [46], that motor tasks performed in virtual reality can improve as a result of training [47] and, importantly, that natural (real-world) motor tasks can improve as a result of training in virtual reality [21]. It remains much less clear, however, whether virtual reality is suitable for testing and training decision making. In particular, our experiences have suggested that there is only limited success when testing decision making with regular video, 360-degree video or animated (reconstructed) match situations produced in virtual reality. In each situation, we and/or the players we tested (particularly the highly skilled players) noted shortcomings that fell short of testing natural decision making in some way. These observations led us to better evaluate the likely success of testing and training decision making in virtual reality, and to explore the technological challenges that need to be overcome for virtual reality to reach what we consider to be its true potential for decision making.

Hadlow et al. [48] recently outlined a framework—based on representative learning design [49]—that was devised to predict the degree to which training tasks would improve perceptual skill more broadly in the performance environment. Termed the *Modified Perceptual Training* (MPT) framework, it proposed that the efficacy of the training approach could be predicted based on the degree to which the (i) perceptual function being trained, (ii) the stimulus and (iii) the response during training matched those used during competition. Virtual reality was used by Hadlow et al. [48] as an example of a task that trained perceptual–cognitive functions that were well matched to those relied on during competition (i.e. anticipation and decision-making skill). However, the rating of the stimulus in virtual reality was only modest and was rated as poorer than that available from other sport-specific stimuli such as video- or image-based training tasks. Finally, the participant response in virtual reality was rated very highly given that responses can closely match those performed in competition. Overall, the MPT framework suggested that virtual reality holds promise as a means of improving competitive sport performance, but that the stimulus was the greatest barrier to efficacy.

Here, we wish to extend on the MPT framework’s evaluation of virtual reality, and especially to dive deeper into the factors likely to influence the representativeness of the stimulus in virtual reality. Hadlow et al. [48] rated the stimulus in virtual reality as being poorer than that available in other perceptual–cognitive training tools such as video- and image-based training (where stimulus representativeness was rated as almost perfect), presumably because of the as yet limited nature of the animations used in virtual reality. Instead, here we contend that the representativeness of the stimulus in virtual reality, if designed correctly, has the potential to be considerably better than those used in other forms of video- and image-based training and that Hadlow et al. perhaps over-rated the quality of the stimulus in video- and image-based footage. Video footage has a critical limitation in that it is not possible to present the interactions that occur between the decision maker and each of their teammates and opponents. As we have sought to explain in our framework of decision making, these interactions may represent some of the most critical information required in a decision-making scenario. Virtual reality can, in contrast, account for these interactions and so it offers specific advantages over other stimuli if modelled correctly. To do so, from the perspective of the MPT framework, a consideration of the potential of virtual reality needs to account for the interactions between the stimuli and the response characteristics within the training environment.

### Existing Approaches for Training Decision Making in Virtual Reality

It is already possible to train decision making in virtual reality, though we contend that the efficacy of these approaches remains limited. Here, we focus on what are probably the two most common forms of virtual reality training presently used in invasive team sports: reconstructed match situations and isolated drills.

Reconstructed match situations refer to virtual reality environments that use computer animations to recreate entire matches (or parts thereof) in which players can be embedded. Using tracking data from the match (i.e. the on-field coordinates of each player and the ball recorded using video tracking or some other form of player-tracking system), the match is reconstructed so that a participant can take the viewpoint of any player who took part in that match [16]. Say, for instance, that the player tracking data were available for the 1997–1998 NBA Playoffs between the Chicago Bulls and Utah Jazz, that match could be reconstructed in virtual reality and an observer could take the viewpoint of any player who played within that match. In one possible application, these reconstructed matches can be used to put a specific player ‘back into the match’. If, for example, Michael Jordan made a poor decision within the match while playing for the Bulls (as unlikely as that might be) by passing to a marked player and missing an open player at the top of the key, then using a virtual reality headset, Jordan could be put back into that same situation to see how he missed the unmarked player, and what he could do differently next time if in a similar situation. Another possible application is that other players could adopt Michael Jordan’s viewpoint in that game to test what decision they would have made in that same situation and/or to use the scenario to train their own decision making. Similar to what would occur when using video-based decision-making testing or training, the scenario could be played and occluded at, or immediately prior to, the moment of the critical decision to probe the ability of others in that given scenario.

From a decision-making perspective, the reconstructed match situations in virtual reality offer new advances but also possess critical limitations. In particular, the reconstructed matches offer advantages over existing video-based decision-making paradigms (including both regular video [8, 26–28], and 360-degree video footage [50–52]). Some video-based scenarios are limited in that they are not shown from a first-person perspective. For those that are from a first-person perspective, they are typically shown from a stationary viewpoint, as opposed to the moving viewpoint most players experience on field. Another limitation is that the scenarios when filmed are often simplified forms of what would be experienced in real matches (e.g. using small-sided games) or are ‘acted out’ by actors rather than being filmed during actual matches. In contrast, observers when viewing reconstructed matches in virtual reality can adopt the first-person perspective viewpoint of players in actual matches, ensuring that the scenarios are more realistic and at game-level intensity. However, the most vital limitation is that the reconstructed scenarios present a mismatch between the observer’s movement and what they see in the virtual environment. Because the observer adopts the viewpoint of a player who took part in the actual match, that viewpoint will move according to the player’s in-match movements irrespective of whether the observer themselves moves. Accordingly, the movements of the observer are ignored. This decoupling of perception and action violates one of the key principles in our framework of decision making: the observer is not able to interact with others seen within the virtual environment and so the observer is reduced to being a passive onlooker rather than an active agent interacting with their teammates and opponents. In essence, the situation is similar to what is experienced in a video-based scenario. Another key limitation of the reconstructed match situations is that the match is reconstructed using player tracking data only and so the more subtle movements of the players in the match, such as their upper body and head directions, can only be assumed and not necessarily faithfully reconstructed. The absence of this contextually rich kinematic information negates the ability of observers to anticipate the actions of others. Finally, the use of occlusion for decision making continues to negate the ability of excellent decision makers to delay their decision and to create newer more promising options.

Isolated drills are a second type of virtual training scenario currently being employed to improve performance in invasive team sports. These environments place observers into situations that replicate typical training drills (e.g. [53]). This can include drills to hit/throw/kick a ball into a goal, through to drills where the ball must be passed to one of several teammates, thus incorporating an element of decision making. A key advantage of this approach is that the viewpoint seen by the observer matches their movements: if the observer moves, then their viewpoint will change commensurate with those movements. Moreover, the actions of the observer are tracked to afford some degree of feedback on the basis of their actions. In particular, motion trackers can be attached to the hands and/or feet of the observer so that they can ‘pass’ or ‘kick’ a virtual ball in the environment [54]. By tracking the velocity of the movements, algorithms can be used to predict how hard and in what direction the ball would have been passed/kicked, and to then model the subsequent trajectory within the virtual environment [55]. This allows the observer to interact with the environment and to see the consequences of their actions. However, the simple nature of the drills in these tasks leaves considerable room for improvement. Foremost is that meaningful interactions with other players (both teammates and opponents) are still typically lacking. Although other players may be visible within some of these isolated drill-based scenarios, those players do not typically respond to the movements of the observer. Accordingly, the meaningful windows of opportunity that are created by virtue of the interactions between the ball carrier and the other players are still lacking. In addition, these isolated drills are situations that are typically trained outside of the game context, and so the degree to which any skills learned might transfer to a game situation remains in question.

### Design Principles for Representative Decision Making in Virtual Environments

**Fig. 3:**
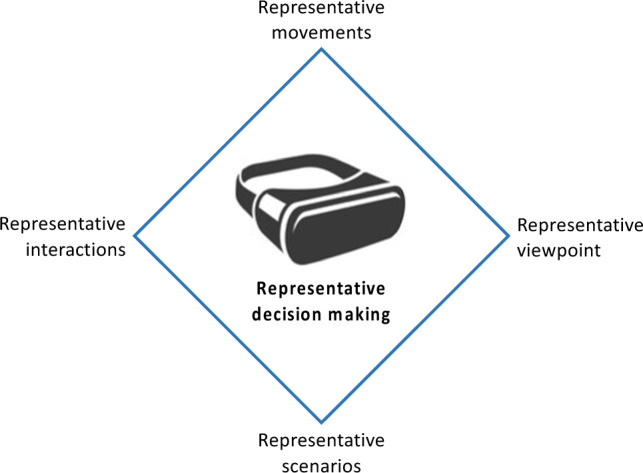
Key design principles for representative decision making in virtual and augmented environments. Representative decision making will require representative movements, viewpoints, interactions and scenarios

Here, we seek to outline design principles for virtual environments that will, in the future, more effectively test and train decision making in invasive team sports. Based on our proposed framework of natural decision making, it highlights four key design features that, if achievable, would provide what we refer to as representative decision making, that is, decision making that reflects that made naturally in the performance environment. In essence, the principles (see Fig. 3) suggest that representative decision making requires representative (i) movements, (ii) viewpoints, (iii) interactions and (iv) scenarios within the virtual environment. Here, we seek to elaborate on each element, how it could be achieved in the future, and the consequences if an element is not realized.

#### Representative Movements

Movements are highly desirable when making decisions [9, 34], yet they are challenging to account for in virtual environments. First, many existing virtual environments rely on observers to move within relatively small, calibrated spaces so that their position can be tracked using registration systems (e.g. HTC Vive Lighthouses that typically require observers to remain within a 5 × 5 m area for registration). Moreover, headsets are sometimes tethered to a computer using cables. The necessity to remain within these confined spaces limits the degree to which observers can currently run and/or move. Solutions are preferable where a headset does not need to be tracked using an external camera system and/or that afford much more space in which to move. Second, representative decision making will necessitate the ability to accurately detect the type of sport-specific kinematic actions that the observer might rely on to interact with their environment (e.g. a throw, kick or pass). Movements can be registered, for example, by using wearable trackers such as inertial sensors and/or using video-based image recognition. Irrespective of the approach, representative decision making will require the virtual environment to be able to detect the movement and to accurately predict and provide the consequences of that action in real time (albeit via visual, haptic and/or auditory feedback).

What would happen if training took place in an environment where the participant response poorly represented the movement performed in the natural environment? We speculate that training benefits are still possible (e.g. [56–58]), but that training will improve only a subset of the skills necessary for optimal decision making on field. Decision making in the absence of movement will fail to foster the embodied decision making that skilled athletes are presumed to rely on [59, 60], where decisions lead to actions, but also actions lead to decisions. Without a movement, the learner also misses crucial feedback about the outcome of their decisions given that they will not be able to see the consequence(s) of their decision. Little risk is associated with performing this at training, though athletes will not learn how their own movements help to open up new decision-making opportunities (i.e. by creating space or running past an opponent). A riskier proposition is to train in environments that provide false or poor-quality feedback about the outcome of a movement. Doing so could result in the athlete attuning to information that is different to that which they would rely on on-field, seriously limiting the degree to which the skill learned in the virtual environment will transfer to skill in the natural environment [48, 49], or worse still, even resulting in negative transfer where the real-world skill becomes worse as a result of virtual-reality training.

#### Representative Viewpoint

Representative decision making will require the observer in virtual reality to adopt a viewpoint which matches that they would experience on field. In a simple sense, a representative viewpoint should be from an egocentric (first person) perspective that faithfully replicates the on-field perspective, rather than the allocentric perspective relied on when observing typical video or television broadcast footage [61]. Crucially, the viewpoint should change commensurate with the movements of the observer. Unlike the present situation when using video footage or reconstructed match situations in virtual reality, the observer should be able to control their own viewpoint by virtue of their own movement so that they can explore by moving within the environment to learn about the consequences of their actions. This representative viewpoint is necessary to facilitate the type of interactions that we propose are necessary for representative decision making. A less-representative viewing perspective will decouple perception from the actions of the person being trained, again limiting the ability of the learner to exploit their movements to open up new decision-making opportunities.

Realistic animations are also a vital ingredient for providing a representative viewpoint to participants in a virtual environment. Representative decision making will require the observer to pick up, for instance, information that might help them to anticipate the direction in which others are running, including the gaze direction of defenders [62], and so relatively high levels of computer animation are desirable to provide the level of visual fidelity necessary to make this type of information available to the observer. At present, the use of animations represents a disadvantage of virtual reality when compared with, for instance, video footage, which displays natural, real-world footage [63]. Present systems are typically lacking this level of detail in their animations. However, if this limitation were to be rectified in the future (i.e. through advances in animation), then virtual reality will offer additional advantages, including the opportunity to manipulate the appearance of players to, for instance, examine if and how decision-making behaviour differs according to changes in contextual information related to the appearance of teammates and opponents (e.g. see [64]). Less-representative animations in virtual reality will likely result in decisions that are delayed, given that faithful kinematic information will not be available on which to make earlier decisions [33], and/or may train learners to attune to different (i.e. non-kinematic) sources of information than what would be typically used on field.

#### Representative Interactions

A key tenet of our framework of decision making is that representative decision making will require interactions between the observer and both their teammates and opponents. It is not enough for players to simply follow pre-determined trajectories as they do in video-based tests or in reconstructed match situations; instead, realistic scenarios will require players seen within the environment to alter their trajectories based on the actions of others. As our decision-making example from FC Barcelona shows, a3's off-the-ball and then on-the-ball movements had consequences for the actions of others (not just his immediate defender). Similarly, a representative virtual environment should replicate this behaviour. The movements of the players seen in the virtual environment should change in realistic ways according to the movements of the observer placed into the environment.

Representative interactions are not a trivial challenge to overcome when designing a virtual environment. Having effectively tracked the movements of the observer, it is necessary to then predictively determine if and how those actions might influence the movements of others. The movements of the immediate defender (or the person they are defending against) can be predicted using existing models of one-on-one dyadic interactions based on behaviour observed in invasive team sports [65]. When determining if and how the movements of others might be influenced, data science models built on the basis of player tracking data may be used to control those movements [31, 66, 67]. Having determined where the players should move, the subsequent challenge is to faithfully animate those actions. Again, this is not a trivial task given that those movements may be unique. New advances in neural networking (motion matching) techniques provide exciting opportunities for producing appropriate animations for even novel movements [68].

We contend that training in environments without interactions (or with less-representative interactions) will improve the ability to make passive decisions, without learning how to manipulate opponents and space to generate decision-making opportunities. Good decision makers learn to create opportunities, and training in the absence of interactions will miss this vital element of decision making. Essentially, it will entail little risk and result in the type of training improvements currently possible using video training [29].

#### Representative Scenarios

Decision making is typically tested and trained by participants making judgements when placed into discrete scenarios (i.e. small subsections of a match), rather than taking part in an entire match itself (e.g. [26]). This approach aids to maximize the number of decisions required of a player in a set amount of time and can help in providing discrete repeatable situations in which the most appropriate decision(s) are known. However, the scenarios are not always entirely representative of the in-match situations they are designed to replicate, e.g. if they are acted out and/or include fewer players than would be present in a regular match. Furthermore, there might be only a discrete number of scenarios available. The decision-making scenarios that observers are embedded into in virtual reality should be representative of those that they might experience in real matches and should reflect the role that they specifically would play in the natural environment [69]. Here, virtual reality has much to offer, with the possibility to demonstrate any scenario imaginable instead of the type of fabricated or ‘acted out’ scenarios used in many video-based tests of decision making. Ideally, player tracking data can be used to learn about and programme the most likely scenarios, but also to produce variants of that scenario to facilitate ‘repetition without repetition’ [21]. If a player struggles with a particular scenario, either in a match or in the virtual environment, pattern-matching algorithms can be used with large databases of tracking data from actual matches to recognize and recreate similar scenarios [70]. In this sense, players can be placed into realistic scenarios that originate from actual matches but adapt organically to the movements of the decision maker being trained.

Decision making could indeed be trained using less-representative scenarios, and would involve little risk, but we would expect doing so to detrimentally impact the quality of on-field transfer. Training with less-representative scenarios will mean that the athlete trains in situations that they are less likely to face during competition, for instance when a back trains in situations designed for a forward, or at speeds of play that are much faster/slower than those experienced during competition (e.g. by training females in scenarios showing male players, or vice-versa; [29]). In contrast, representative scenarios will allow a more tailored approach to decision-making training that adapts the training design to the constraints faced by the athlete on field, and that can address the specific weaknesses of that athlete.

Here, we have set out the ideal requirements for an environment that facilitates representative decision making, in addition to what is likely to occur if training when each of the design principles is compromised. But should decision making still be tested and/or trained when an environment does not meet each of these requirements? We argue yes, but that doing so will train only a subset of the skills required for natural on-field decision making. Crucially, we suggest that the consequences are greater for *training* in less-representative environments than they are for testing decision making. When testing decision making, a less representative test will likely underestimate the true nature of the expert advantage. For instance, experts consistently outperform less-skilled players in studies of video-based decision making [7, 71], despite the fact that those tests lack several of the factors we here propose to be important (e.g. interactions, first-person perspective and/or realistic scenarios). Similarly, a virtual environment that lacks true representativeness (e.g. excludes interactions) will likely under-represent the true magnitude of the expert advantage when testing skilled athletes. Skilled performers will likely outperform others on the task, but the test will miss for instance their ability to co-conspire with teammates to create opportunities through interactions. It may be that these more representative tests are better able to differentiate the decision-making ability of athletes of similar abilities (e.g. between elite and subelite athletes), rather than only differentiating between elite and novice athletes as is often the case with existing tests of decision making [36, 50].

It remains unclear whether training in a less-representative environment will result in improved on-field performance. Transfer should occur if the virtual training allows the learner to attune to information that would be relied on on-field [48], for instance to better pick up information about the movements of teammates relative to their defenders, or to familiarize themselves with new defensive structures. However, the degree of transfer may be compromised if the learner is for instance not able to move while making those decisions, because the nature of the visual information will be different to that they would rely on when moving on field [59, 60]. As noted earlier, the greatest risk in training in a virtual environment is likely to exist if the scenario misrepresents the information that would be relied on naturally and requires the learner to attune to less functional or even non- or dysfunctional information [72]. Examples include if the nature of the anticipatory information is different to that found on field, or if the available feedback about an action outcome differs to that offered in the natural environment (e.g. if a pass is seen to be successful in the virtual environment when it would not have been on field). In those instances, the learner may learn to rely on information that is absent or even misrepresents that available naturally on field.

## Conclusions

Virtual and augmented reality offer exciting opportunities for sampling the natural decision-making skill of invasive team-sport athletes, but the degree to which those environments might be successful for both testing and training decision-making skill has remained unclear. In this paper, we have attempted to fill this gap by outlining a framework for natural decision making in invasive team sports, and to outline, on the basis of that framework, the design principles necessary for testing and training representative decision making in a virtual environment. Natural decision making was conceptualized as a cyclical process whereby ‘windows of opportunity’ are created on the basis of interactions between the ball carrier and their teammates and opponents, with decisions made both with and without the ball. Moreover, anticipation was considered a subset of decision making, with accurate decisions requiring the observer to constantly anticipate the actions of others based on both kinematic and contextual information. Currently, limitations of existing virtual reality environments mean that some of these important aspects of decision making remain absent, in particular, the ability to interact with others and to anticipate the actions of others. Based on this, we outlined a framework of four specific design principles that should be met to optimally test and train decision making in a representative way in virtual environments, including the necessity for representative movements, viewpoints, interactions and scenarios. Future technological developments in each of these four areas will hopefully allow virtual and augmented reality to fulfill their promise of providing new and exciting ways to improve the on-field decision making of invasive team sport athletes.

## Declarations

### Permissions

In preparing this paper, every effort has been made to respect all intellectual property rights of third parties and, where necessary or possible, seek permission to use their work.
